# A One‐Step Approach to the Synthesis of High Aspect Ratio Titania Nanoflakes

**DOI:** 10.1002/gch2.201700060

**Published:** 2017-10-11

**Authors:** Scott Brown, Hassan El‐Shall, Yang‐Yao Lee

**Affiliations:** ^1^ 205 Particle Science & Technology University of Florida Gainesville FL 32611 USA

**Keywords:** high aspect ratio, modified surface hydrolysis, nanoflakes, one‐step synthesis, titanium dioxide

## Abstract

High aspect ratio TiO_2_ nanoflakes are synthesized by a one‐step modified surface hydrolysis method. Surface morphology and physical dimensions are characterized using scanning electron microscopy, laser diffraction analysis, and transmission electron microscopy. Microsized flakes having a thickness ≈40 nm are successfully synthesized by spreading an oil phase consisting of titanium tetraisopropoxide and a low surface tension hydrocarbon on the surface of water. Pure anatase phase crystalline titania nanoflakes are obtained by calcining at 400 °C without changing the shape and thickness of flakes. Relatively higher specific surface area (2–6 times) and less crystal defects enhance photocatalytic activities of nanoflakes due to more surface reaction sites and the suppression of fast recombination. By performing dye degradation under ultraviolet illumination, titania nanoflakes exhibit the higher photocatalytic efficiency over the commercial photocatalyst, Degussa P25. As far as it is known, this method is the most efficient and cost effective process for making low‐dimensional nanomaterials in a continuous manner. These titania flakes can be easily separated from the treated water by simply sedimentation or filtration and therefore is very suitable for water purification application.

Nanostructured titanium dioxide has attracted tremendous interest in the field of environmental purification, solar energy conversion, pigment, optics, gas sensing, and energy storage because of their unique physicochemical properties.^1^, ^2^ Many scientific works have been focused on particle size down to the order of nanometer. However, using TiO_2_ nanoparticles for water treatment is limited in practical application since it is very difficult to be removed from purified water due to very small mass. The conventional separation methods such as centrifuging, filtration, and sedimentation cannot be used to solve this problem efficiently and economically. Except for size control, shape control of particulates on nanometer scale is more difficult and starts to attract attention recently. Many nanomaterials, more specifically anisotropic nanostructures, have been successfully synthesized including nanotubes, nanowires, nanorods, nanofibers, and nanosheets.^3^, ^4^, ^5^, ^6^, ^7^, ^8^, ^9^, ^10^, ^11^ However, most synthesis methods for these types of particles require multiple, complicated synthesis procedures and are typically nonconductive to be scaled up manufacturing as yields are typically milligram or less quantities of material. Examples of this includes template, chemical vapor deposition, hydrothermal, electrochemical anodization, etc.^12^, ^13^, ^14^, ^15^

Almquist and Biswas prepared anatase powders with sizes ranging from 5 to 165 nm employing various synthesis methods and found that an optimum particle size range of 25–40 nm within all photocatalytic experiments regardless of fabrication processes.^16^ The authors suggest that the optimum particle size is a function of several competing mechanisms such as light absorption and scattering efficiency of the particles, as well as electron–hole pair combination and interfacial charge transfer. Therefore, cost effective and mass production available synthesis for titania nanomaterials with at least 1D close to the optimum size of photocatalyst will be a revolution of the whole titania fabrication.

In this study, micrometer sized titania flakes having a thickness ≈40 nm were successfully synthesized by spreading an oil phase consisting of titanium tetraisopropoxide and a low surface tension hydrocarbon on the surface of water.^17^ These nanoflakes were produced by restricting the hydrolysis of titania precursor within dimension of the organic thin film due to the difference of surface energy to water. The flakes were also calcined at 400 °C resulting in high purity anatase having near identical dimensions to the uncalcined material. Investigations have shown the described method to be efficient and economical at producing gram to kilogram quantities of material. This study examines these nanoflakes which were characterized by scanning electron microscopy (SEM), laser diffraction analysis, X‐ray diffraction (XRD), transmission electron microscopy (TEM), physisorption techniques, and UV–visible spectrometer.

The surface morphology of synthesized titania nanoflakes was investigated using SEM images, shown in **Figure** **1**. The major diameter of the flakes was found to be of the order of 10–20 µm (Figure 1A,B), and the thickness of these flakes was ≈40 nm (Figure 1C,D). The flakes were further treated by calcination at 400 °C for 2 h. Aggregation was not apparent when comparing these treated flakes (Figure 1B) with those obtained directly from synthesis (Figure 1A). Moreover, the thickness of calcined flakes did not change by the heat treatment (Figure 1D). Both the uncalcined and calcined flakes were transparent under the SEM (Figure 1B).

**Figure 1:**
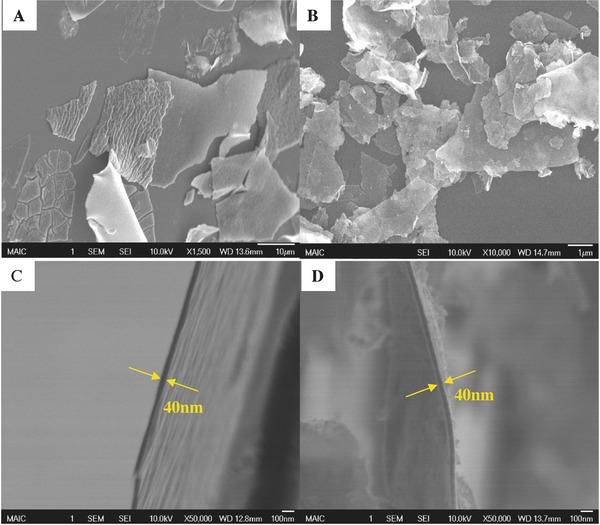
SEM images of titania nanoflakes: A) synthesized samples, B) calcined samples, C,D) edge views of (A) and (B), respectively. Images were obtained without conducting coating.

Particle size distributions of synthesized and calcined flakes were measured using laser diffraction. Laser diffraction particle size analysis alone is unsuitable for characterizing anisotropic particles as the analyzer assumes the particles conform to a spherical model. In combination with electron microscopic method it is possible to garner a better understanding of the synthesized particles morphology. The measured major dimension of the flakes was comparable to the dimensions obtained from the SEM images (Figure 1A,B). The statistics data of particle size distribution by volume were shown in **Table** **1**. The synthesized material has abroad size distribution spanning from 1 to 100 µm with a *D*_50_ of 20.8 and 19.0 µm for the synthesized and calcined flakes respectively (**Figure** **2**). Comparing the volume distributions it is evident some larger flakes break or crack during dehydration and crystallization. The average aspect ratio of the flakes is 1:500 which can be calculated from mean size (Table 1) divided by thickness.

**Table 1:** Particle diameter statistics for synthesized and calcined titania flakes under investigation

Sample	*D*_10_ [μm]	*D*_50_ [μm]	*D*_90_ [μm]	Mean [μm]	Standard deviation [μm]
Synthesized flakes	5.2	20.8	81.6	39.1	58.9
Calcined flakes	5.1	19.0	51.8	24.7	20.9

**Figure 2:**
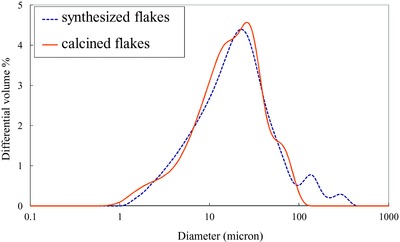
Volume based particle size distribution for synthesized and calcined titania flakes.

Crystalline structure changes of the titania flakes was monitored by powder XRD (**Figure** **3**). The synthesized flakes show broadening and weak Bragg peaks which indicates the nature of flakes consist of partial amorphous material with a presence of the anatase phase (Figure 3A). After heat treatment, the expected phase transformation from amorphous to crystalline titania was confirmed by seven characteristic diffraction peaks (Figure 3B). The heat treated flakes were converted to a pure anatase phase [JCPDS: 21‐1272] which is the most photoactive phase of titania.^18^ There was no indication of the rutile phase by XRD. The intensity of characteristics peaks was comparable to the commercial pure anatase standard materials (Figure 3C) which indicate nearly complete conversion of the amorphous titania. The average crystal grain size can be calculated by the Scherer equation

(1) $$d=\frac{k\lambda }{B\cos\theta_{B}}$$

where *d* is the calculated grain size, λ is the wavelength of X‐ray (Cu Kα 1.54 Å), *B* is the full‐width at half‐maximum intensity, and *θ_B_* is the half of the diffraction peak angle. The grain size was determined to be 4 and 9 nm for synthesized and calcined flakes respectively (**Table** **2**).

**Figure 3:**
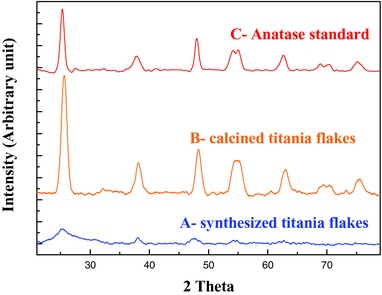
XRD patterns of synthesized and calcined titania nanoflakes.

**Table 2:** Grain size calculation by the Scherer equation for both nanoflakes

Sample	*θ_B_* [°]	*d* [nm]
Synthesized flakes	25.91	4.1
Calcined flakes	25.35	8.7

High resolution transmission electron microscopy (HR‐TEM) images show the flakes are comprised of circular crystalline platelets of about 5–8 nm in diameter. (**Figure** **4**A,B). The interference lattice fringes can be seen in the TEM images and had a separation distance of 0.35 nm, corresponding to the interplanar spacing of the (101) planes for anatase.^19^ Random orientation of individual grains over the both flakes was suggested from the concentric diffraction rings in the select area diffraction mode and consistent with the anatase (101), (004), (200), (105) for circles 1 to 4, respectively (the insets of Figure 4). On closer inspection, an amorphous layer can be seen surrounding the smaller circular crystallites in synthesized sample (Figure 4A). After calcination, some pores were developed due to local rearrangement and growth of crystal grains. Consequently, it is thought that the flakes are polycrystalline and consist of fine grains of anatase.

**Figure 4:**
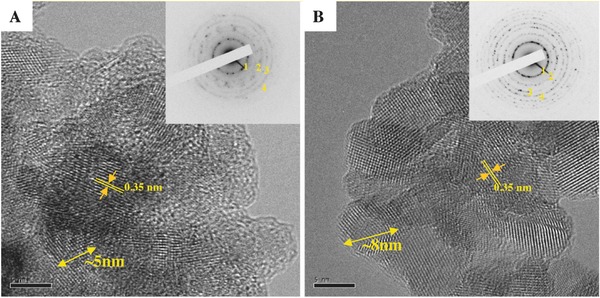
HR‐TEM images of titania nanoflakes (the SAD pattern as inset). A) synthesized samples; B) calcined samples The diffraction rings are indexed as (1) 101, (2) 004, (3), 200, (4) 105 for anatase.

The specific surface area of synthesized and calcined titania nanoflakes was estimated using nitrogen absorption isotherms in conjunction with the Brunauer–Emmett–Teller (BET) model. The high specific surface area of titania flakes were expected and attributed to high aspect ratio (250:1 to 500:1) and averaged 5–8 nm fine grains throughout the surface of flakes. In addition, several 2D titania nanomaterials were revealed having high specific surface of 110 to 320 m^2^ g^−1^, respectively from exfoliation of a layered precursor^6^ and lamellar micelle template.^20^ It is desirable for photocatalytic materials to have higher specific surface area as this usually results in higher photoactivity since a large amount of adsorbed organic molecules at surface sites, increasing the reaction rate. However, high surface area powders are usually associated with large amounts of crystal defects favoring fast recombination of electrons and holes, ultimately leading to lower photoactivity. Compared to a commercial photocatalyst, Degussa P25, the surface areas of nanoflakes were 2–6 times higher (**Table** **3**). The photocatalytic activity of these flakes was investigated by performing dye degradation experiments under ultraviolet activation.

**Table 3:** Physisorption measurements of P25, synthesized and calcined titania nanoflakes

Sample	Specific surface area [m^2^ g^−1^]	Specific pore volume [cm^3^ g^−1^]
Synthesized nanoflakes	323	–
Calcined nanoflakes	110	0.342

**Figure** **5**A shows typical UV‐visible diffuse reflectance spectra in the wavelength range of 250∼500 nm for the synthesized flakes together with the calcined flakes. The sharp decrease in the diffuse reflectance in the UV region results from the fundamental light absorption of the semiconductor materials and a blue shift of the onset of reflectance occurred at calcined sample due to heat treatment. In semiconductor physics, the general relation between the absorption coefficient and the band gap energy is given by

(2) $${\left(\alpha h\nu \right)}^{m}=h\nu -E_{g}$$

where *m* is an index depending on the nature of the electron transitions, α is the absorption coefficient, *h* is the Planck constant, ν is the frequency of electromagnetic radiation, and *E* is band gap energy of the semiconductor. To estimate band gap energies of both nanoflakes, α data in Figure 5A was plotted as (*αhν*)^2^ versus *hν* and the optical absorption energy was determined via extrapolation. An increase in band gap from 3.25 to 3.33 eV, i.e., a blue shift, is mostly likely due to the quantum confinement effect of higher crystallinity after calcination and thin flaky morphology.^21^ The atomic structure at the grain boundary is different from that in the amorphous area, leading to larger free carrier concentrations and the existence of potential barriers at these boundaries. Therefore, an electric field is built up, the band gap energy increases, and the absorption limit shifts to shorter wavelength range.

**Figure 5:**
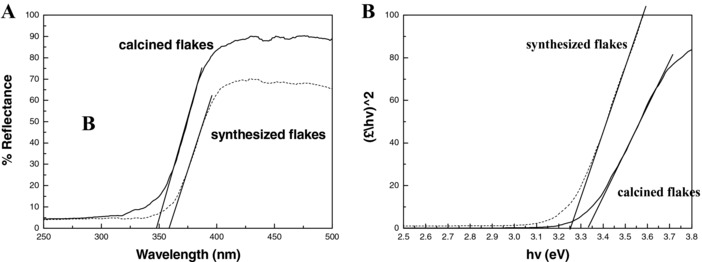
A) Diffuse reflectance spectra and B) the dependence of (*αhν*)^2^ on the photon energy for synthesized and calcined titania flakes.

**Figure** **6** compares the photocatalytic activity of P25, synthesized and calcined titania nanoflakes by removing the methylene blue form water under UV light irradiation. Degussa P25 was used as a reference material. It has a mean diameter of 30 nm which is comparable to the minor dimension of nanoflakes (40 nm). It was observed that calcined flakes exhibited the highest photocatalytic efficiency on the degradation of methylene blue among all tested samples. Besides, titania flakes in this study demonstrated not only higher photocatalytic activity on removing methylene blue from water but the easier fabrication against the state of art titania nanomaterials such as nanofibers, nanotubes, nanocomposite.^22^, ^23^, ^24^, ^25^ The main photocatalysis reactions were identified by laser flash photolysis and listed as follows^26^

-  Charge–carrier generation
  (3) $${\mathrm{TiO}}_{2}\;+\;h\nu \;\to \;h_{\mathrm{VB}}{}^{+}\;\;+\;e_{\mathrm{CB}}{}^{-}\;\;,\ldots \ldots \ldots 10^{-15}s$$
-  Charge–carrier trapping
  (4) $$h_{\mathrm{VB}}{}^{+}\;\;+>{\mathrm{Ti}}^{\mathrm{IV}}\mathrm{OH}\to {\left\{>{\mathrm{Ti}}^{\mathrm{IV}}\mathrm{OH}\;•\right\}}^{+},\ldots \ldots \ldots 10\;\times \;10^{-9}\;s$$
  (5) $$e_{\mathrm{CB}}{}^{-}\;\;+>{\mathrm{Ti}}^{\mathrm{IV}}\mathrm{OH}↔{\left\{>{\mathrm{Ti}}^{\mathrm{III}}\mathrm{OH}\right\}}^{-},\ldots \ldots \ldots 100\;\times \;10^{-12}\;s$$
  (6) $$e_{\mathrm{CB}}{}^{-}\;\;+>{\mathrm{Ti}}^{\mathrm{IV}}\to \;>{\mathrm{Ti}}^{\mathrm{III}},\ldots \ldots \ldots 10\;\times \;10^{-9}\;s$$
-  Charge–carrier recombination
  (7) $$e_{\mathrm{CB}}{}^{-}\;\;+{\left\{>{\mathrm{Ti}}^{\mathrm{IV}}\mathrm{OH}\;•\right\}}^{+}\to \;>{\mathrm{Ti}}^{\mathrm{IV}}\mathrm{OH},\ldots \ldots \ldots 100\;\times \;10^{-9}\;s$$
  (8) $$h_{\mathrm{VB}}{}^{+}\;\;+{\left\{>{\mathrm{Ti}}^{\mathrm{III}}\mathrm{OH}\right\}}^{-}\to \;>{\mathrm{Ti}}^{\mathrm{IV}}\mathrm{OH},\ldots \ldots \ldots 10\times 10^{-9}\;s$$
-  Oxidation or reduction
  (9) >TiIVOH •++ Red0→ >TiIVOH + Red •+,………100 × 10−9 s
  (10) $$e_{\mathrm{TR}}{}^{-}\;\;\;+\;O_{x}\to \;>{\mathrm{Ti}}^{\mathrm{IV}}\mathrm{OH}+O_{x}{•}^{-},\ldots \ldots \ldots 10^{-3}\;s$$

Where $e_{\mathrm{CB}}^{-}$ is a conduction band electron, $h_{\mathrm{VB}}^{+}$ is a valence band hole, >TiOH represents the hydrated surface of TiO_2_, {>Ti^IV^OH •}^+^ is the surface‐trapped hole, {>Ti^III^OH}^−^ is the surface‐trapped electron, Red is an electron donor (i.e., reductant), O_x_ is an electron acceptor (i.e., oxidant). From the above reactions, highly reactive species such as hydroxyl radicals (OH•) and superoxide ions (O_2_ •^−^) could either oxidize or reduce wide variety of organic compound in waste water. The possible degradation mechanism of methylene blue (MB_abs_) through photocatalysis is the formation of intermediates (MB_abs_*)such as azure A, B, and C dyes and thionin by cleavage of one or more of the methyl groups substituent on the amine groups.

(11) $${\mathrm{MB}}_{\mathrm{abs}}\;+\;\mathrm{OH}\;•\;\to \;{\mathrm{MB}}_{\mathrm{abs}}{}^{*}$$

**Figure 6:**
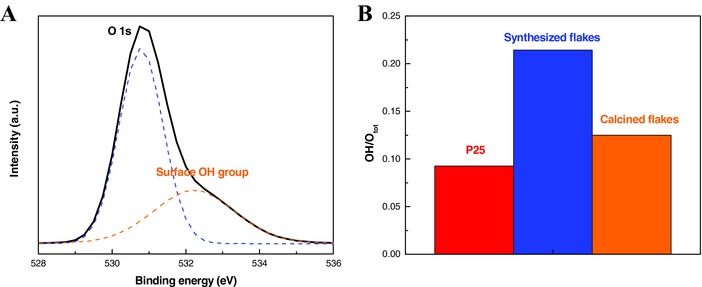
A) XPS O 1s peak fitting of synthesized flakes. B) OH/Otot XPS surface ratio of the titania samples.

These colored molecules were identified and reported by LC–MS technology during the through the demethylation cleavage during the photocatalytic degradation.^27^, ^28^, ^29^ However, the total mineralization of methylene blue was proved by completed decoloration after photocatalysis of titania flakes. Except for specific surface area, the surface chemistry of titanium dioxide is another surface property which significantly affects the photocatalytic reaction. It was well known that the chemistry of metal oxides surface is mainly dominated by the hydroxyl groups arise from the interaction with the environment such as moisture in atmosphere or water in aqueous solution.^30^, ^31^ Surface OH groups were determined by taking XPS spectra of O 1s signal for all of the samples under the same preparation condition. Using the Gaussian mixture peak fitting technique, two components are shown in the typical O 1s XPS spectrum (Figure 6A). One represents the oxygen element in titania lattice (529.9 eV) and the other corresponds to the surface hydroxyl species (531.9 eV).^32^ The ratio of surface OH to total oxygen 1s signals for the samples is shown in Figure 6B. It should be noted that both samples have higher surface hydroxyl concentration than commercial product, P25, on the same mass basis. Calcined flakes have less OH groups than synthesized samples due to the dehydroxylation process after heat treatment. It has been widely reported that the removal of surface absorbed water was carried out by annealing the sample at 450–500 °C.^30^, ^33^ Higher density of OH groups for synthesized and calcined flakes were attributed to the higher specific surface area comparing to the Degussa P25. Hydroxyl radical, one of the most reactive species in the photocatalysis process, is strongly related to the hydroxyl group concentration at titania surface. Therefore, we could expect that better photocatalytic performance could be achieved by the sample showing the larger OH component in the XPS oxygen 1s signal.

Three titania samples demonstrated similar dye adsorption in the dark within 2 h which suggested photocatalysis is the main reaction instead of physical adsorption (**Figure** **7**A). A first order rate reaction was shown in Figure 7B which indicates dye concentration is the limiting factor. In contrast, significant enhancement was observed when agitation was added to the system in the form of introducing air bubbles into the system continuously (Figure 7C). The reaction with added turbulence presented a pseudo first order fashion and much higher efficiency especially for the flake systems. One possible explanation of these differences may be correlated to oxygen depletion during the photocatalysis process. From the results of XRD and TEM, both flakes consisted of very small nanocrystallites which imply a large amount of defects (grain boundaries) within both materials. Consequently, fast recombination of photoassisted charge carriers preferentially occurs at these local defect sites and dominates the reaction. The flake samples have much higher surface area than P25. However, the photocatalytic performance is not proportional to surface area without supplying oxygen to the system. By introducing air into the system, dissolved oxygen will primarily become an electron accepter and may form superoxide radicals.^34^ Dissolved oxygen adsorbed at titania surface to trap the electrons in order to prevent the harm of charge–carrier recombination through the following reaction

(12) $$O_{2}\;+\;e^{-}\to O_{2}^{•\;-}$$

**Figure 7:**
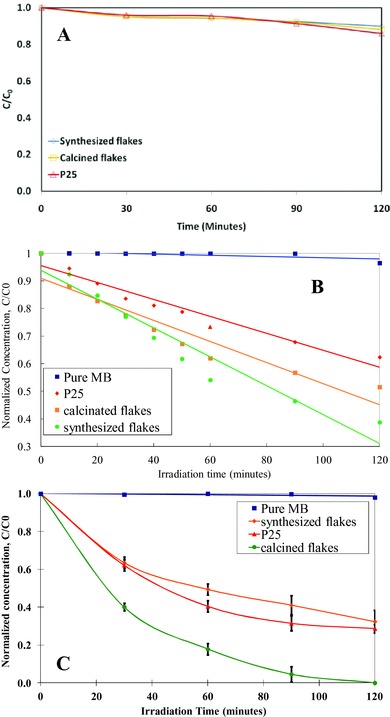
Photocatalytic decomposition of methylene blue by using A) synthesized and calcined flakes, B) dye adsorption in the dark, C) without bubbling treatment with bubbling treatment.

The highly reductive species, superoxide radials, was detected directly by the electron spin resonance technology.^35^ Besides, the partial pressure of oxygen in the solution was revealed to be a vital factor in the heterogeneous photocatalysis.^36^, ^37^, ^38^ Therefore, aeration process remarkably increase the photocatalytic reaction rate through better charge separation and reduction ability. More importantly, fast recombination, the rate limiting step, could be depressed by eliminating excited conduction band electron for higher efficiency.

In summary, a high aspect ratio titania nanoflakes has been synthesized. Compared to other methods for making low‐dimensional nanomaterials, this spreading film process could continuously produce nanoflakes with a cost effective manner. These titania flakes could be easily separated from the treated water by simply sedimentation or filtration and therefore is very suitable for water purification application.

## Experimental Section

*Synthesis*: Titania nanoflakes were fabricated by spreading a mixture of stearic acid (Fisher, 99%), low surface tension hydrocarbon (for example, octane, Fisher, 97%), and titanium tetraisopropoxide (Aldrich, 97%) on the surface of solution consist of high purity water (Barnstead Nanopure Infinity, 18 MΩ cm^−1^), nitric acid, and sodium dodecyl sulfate (Fisher, 99%). Stearic acid and the hydrocarbon were used to decrease the viscosity of titania precursor and enhance the spreadability of the mixture. The thickness of nanoflakes could be manipulated by varying the volume ratio of titania precursor and hydrocarbon. Typically, a ratio of 1:8 of titania tetraisopropoxide to hydrocarbon was used to produce titania nanoflakes with thickness about 40 nm. The resulting slurry was washed with Nanopure water and then centrifuged (Begman BH‐2) at 3000 rpm for 15 min to concentrate the slurry. The particles were then resuspended in isopropanol and centrifuged to remove further impurities. This was repeated a total of five. Finally, the nanoflakes were dried by a supercritical fluid drying process described elsewhere.^39^

*Characterization*: The surface morphology and thickness of titania nanoflakes were observed by a cold filed emission SEM (JEOL 6335). Samples were made by depositing titania slurry on a smooth silicon wafer as substrate and placed to a vacuum oven for drying. XRD patterns were obtained on dried powders using a Philips APD 3720 diffractometer (Cu Kα radiation, λ = 1.54 Å). Particle size distributions were measured with a laser diffraction particle size analyzer (Beckman Coulter LS 13 320). When using a differential volume distribution for flaky particles moving in turbulence pass the laser beam, the maximum diameter was measured through the average random orientation of the flakes. The larger dimension of nanoflakes was estimated by dispersing both flakes in deionized water with liquid modules. HR‐TEM images were obtained using a JEOL 2010F. The specimens were prepared by dispersing materials in isopropanol and placed on a lacy carbon grid. Specific surface area, pore size distribution were evaluated using a 5 point BET analysis with a Quantachrome NOVA 1200.

*Photocatalysis*: Dye decomposition experiments were performed inside a cylinder UV reactor equipped with 6 UVA lamps, RPR‐3500A which emit light in a band between 300 and 420 nm with an approximately Gaussian distribution centered at 350 nm (Southern New England Ultra Violet Company, Branfield, CT). The light intensity of photocatalytic reactor was around 10 mW cm^−2^ monitored using LI‐250a light meter (Model Pyranometer, LICOR, Biosciences, USA). A 150 mL Erlenmeyer flask containing dye solution with TiO_2_ particles at the constant concentration was placed inside the UV reactor and continuously agitated by a magnetic stir. House air was delivered by a glass tip connected to the plastics hoods from air outlet for investigating the aeration process. The amount/volume of input air flow was controlled by a flow meter. A cold air flow generated by a cooling fan circulated through the cylinder UV reactor was to prevent the heating effect during the photocatalysis process. 100 mL of dye solution with concentration of 50 × 10^−6^ m at 100 ppm catalyst loading were prepared. Three photocatalysts were added to the dye solution respectively and then stirred for 10 min in the dark before UV light illuminating. Dye concentrations for photocatalysis experiments were determined using a Perkin‐Elmer Lambda 800 UV–vis spectrometer and diffuse reflectance spectra were taken by the spectrometer coupled with an integrating sphere.

## Conflict of Interest

The authors declare no conflict of interest.

## References

[1] a) S. Iijima , Nature 1991, 354, 56;

[2] a) X. Peng , A. Chen , Adv. Funct. Mater. 2006, 18, 2807;

[3] a) S. Ljjna , Nature 1991, 354, 56;

[4] A. M. Morales , C. M. Lieber , Science 1998, 279, 208. 9422689 10.1126/science.279.5348.208

[5] J. M. Wu , T. W. Zhang , Y. W. Zeng , S. Hayakawa , K. Tsuru , A. Osaka , Langmuir 2005, 21, 6995. 16008414 10.1021/la0500272

[6] T. Sasaki , S. Nakano , S. Yamauchi , M. Watanabe , Chem. Mater. 1997, 9, 602.

[7] I. Moriguchi , H. Maeda , Y. Teraoka , S. Kagawa , Chem. Mater. 1997, 9, 1050.

[8] L. Zhang , D. Jing , X. She , H. Liu , D. Yang , Y. Lu , J. Li , Z. Zheng , L. Guob , J. Mater. Chem. A 2014, 2, 2071.

[9] H. Hou , L. Wang , F. Gao , G. Wei , B. Tang , W. Yang , T. Wu , J. Am. Chem. Soc. 2014, 136, 16716. 25407313 10.1021/ja508840c

[10] H. Hou , F. Gao , L. Wang , M. Shang , Z. Yang , J. Zheng , W. Yang , J. Mater. Chem. A 2016, 4, 6276.

[11] H. Hou , M. Shang , F. Gao , L. Wang , Q. Liu , J. Zheng , Z. Yang , W. Yang , ACS Appl. Mater. Interfaces 2016, 8, 20128. 27430307 10.1021/acsami.6b06644

[12] B. Changdeuck , Y. Hyunjun , K. Sihyeong , L. Kyungeun , K. Jiyoung , M. S. Myung , S. Hyunjung , Chem. Mater. 2008, 20, 756.

[13] V. G. Bessergenev , R. J. F. Pereira , M. C. Mateus , I. V. Khmelinskii , D. A. Vasconcelos , R. Nicula , E. Burkel , Botelho , A. M. do Rego , A. Saprykin , Thin Solid Films 2006, 503, 29.

[14] G. Yupeng , L. Nam‐Hee , O. Hyo‐Jin , Y. Cho‐Rong , P. Kyeong‐Soon , L. Hee‐Gyoun , L. Kyung‐Sub , K. Sun‐Jae , Nanotechnology 2007, 18, 295608.

[15] K. Varghese , D. Gong , M. Paulose , C. A. Grimes , E. C. Dickey , J. Mater. Res. 2003, 18, 156.

[16] C. B. Almquist , P. Biswas , J. Catal. 2002, 212, 145.

[17] Y. Y. Lee , Ph.D. Dissertation, University of Florida, 2010.

[18] a) A. M. Fox , M. T. Dulay , Chem. Rev. 1993, 93, 341;

[19] Y. Q. Wang , G. Q. Hu , X. F. Duan , H. L. Sun , Q. K. Xue , Chem. Phys. Lett. 2002, 365, 427.

[20] P. Kluson , H. Luskova , O. Solcova , L. Matejova , T. Cajthaml , Mater. Lett. 2007, 61, 2931.

[21] T. Sasaki , M. Watanabe , J. Phys. Chem. B 1997, 101, 10159.

[22] M. Nagalakshmi , C. Karthikeyan , N. Anusuya , C. Brundha , M. Jothi Basu , S. Karuppuchamy , J. Mater. Sci.: Mater. Electron. 2017.

[23] M. N. Subramaniam , P. S. Goh , N. Abdullah , W. J. Lau , B. C. Ng , A. F. Ismail , J. Nanopart. Res. 2017, 19, 220.

[24] F. Heshmatpour , S. Zarrin , J. Photochem. Photobiol., A 2017, 346, 431.

[25] T. V. M. Sreekanth , J.‐J. Shim , Y. R. Lee , J. Photochem. Photobiol., B 2017, 169, 90. 28297682 10.1016/j.jphotobiol.2017.03.006

[26] M. R. Hoffmann , S. T. Martin , W. Choi , D. W. Bahnemannt , Chem. Rev. 1995, 95, 69.

[27] C. Yogi , K. Kojima , N. Wada , H. Tokumoto , T. Takai , T. Mizoguchi , H. Tamiaki , Thin Solid Films 2008, 516, 5881.

[28] A. Orendorz , C. Ziegler , H. Gnaser , Appl. Surf. Sci. 2008, 255, 1011.

[29] T. Zhang , T. Oyamaa , A. Aoshimaa , H. Hidaka , J. Zhaob , N. Serpone , J. Photochem. Photobiol., A 2001, 140, 163.

[30] A. Kanta , R. Sedev , J. Ralston , Langmuir 2005, 21, 2400. 15752031 10.1021/la047721m

[31] G. D. Parfitt , Pure Appl. Chem. 1976, 48, 415.

[32] B. V. Crist , Handbook of Monochromatic XPS Spectra: The Elements and Native Oxides, Wiley, New York 2000.

[33] G. D. Parfitt , Progress in Surface and Membrane Science, Academic Press, New York 1976.

[34] A. Houas , H. Lachheb , M. Ksibi , E. Elaloui , C. Guillard , J. M. Herrmann , Appl. Catal. B 2001, 31, 145.

[35] J. Yu , J. Chen , C. Li , X. Wang , B. Zhang , H. Ding , J. Phys. Chem. B 2004, 108, 2781.

[36] M. Barbeni , E. Pramauro , E. Pelizzetti , E. Borgarello , M. Gratzel , N. Serpone , Nouv. J. Chim. 1984, 8, 547.

[37] L. Rideh , A. Wehrer , D. Ronze , and A. Zoulalian , Ind. Eng. Chem. Res. 1997, 36, 4712.

[38] K. H. Wang , Y. H. Hsieh , R. C. Ko , C. Y. Chang , Environ. Int. 1999, 25, 671476.

[39] S. Tedeschi , N. Stevens , D. Cepeda , Y. Y. Lee , K. Powers , M. Ranade , H. El‐Shall , Powder Technol. 2009, 191, 188.

